# Warm acupuncture therapy for Primary sciatica: A protocol of systematic review and meta-analysis

**DOI:** 10.1097/MD.0000000000024551

**Published:** 2021-02-26

**Authors:** Fang Cao, XingWei He, Cui Guo, JingWei Wang, RuiLin Zeng, LunBin Lu, FanLei Meng, Fenfen Zhao, ChangKang Wang

**Affiliations:** a Jiangxi University of Traditional Chinese Medicine; b The Affiliated Hospital of Jiangxi University of Traditional Chinese Medicine, Nanchang; c Liaoning University of Traditional Chinese Medicine, Shenyang, China.

**Keywords:** meta-analysis, primary sciatica, protocols, randomized controlled trials, warm acupuncture

## Abstract

**Introduction:**

This meta-analysis aims to assess the effectiveness and safety of warm acupuncture therapy for treating Primary sciatica.

**Methods:**

The following 9 databases will be search from their inception to December 6, 2020: MEDLINE, EMBASE, the Cochrane Central Register of Controlled Trials (CENTRAL), the Chinese Biomedical Literature Database (CBM), the Chinese Medical Current Content (CMCC), the Wan-Fang Database and the China National Knowledge Infrastructure (CNKI). Randomized controlled trials (RCTs) of warm acupuncture for treating Primary sciatica, Chinese or Japanese without restriction of publication status will be included. Two researchers will independently undertake study selection, extraction of data and assessment of study quality. Meta-analysis will be conducted after screening of studies. Data will be analyzed using risk ratio for dichotomous data, and standardized mean difference or weighted mean difference for continuous data.

**Dissemination:**

This meta-analysis will be disseminated electronically through a peer-reviewed publication or conference presentations.

**Conclusion:**

This study will provide evidence to judge whether warm acupuncture

**Trial registration number:**

INPLASY2020120109.

## 1 Introduction

### 1.1 Description of the condition

Primary sciatica^[1]^ refers to a group of clinical syndromes mainly characterized by pain. The pain is characterized by the appearance of discharge and burning sensation on one or both sides of the waist, posterior hip, thigh and posterolateral leg.^[2,3]^ Some patients may also experience multiregional numbness and paresthesia. Sciatica is a common clinical disease. According to statistics, the incidence of sciatica in China is as high as 11.5% to 13.6%, and it is still increasing year by year.^[3]^ About 60% of them have mild dysfunction caused by mistreatment or other reasons, which seriously affects the quality of life of patients and causes serious social and economic losses.^[4]^ Numerous methods in treatment of root sciatica at present, modern medicine for primary preference for the pathogenesis of sciatica nerve inflammatory reaction, much by teeth, paranasal sinus, tonsil, such as focal infection, caused by the blood and nerve Coat, and more myositis and fibrous tissue inflammation in cold, wet often accompany for inducing factors.^[5]^ Western medicine clinical treatment is divided into surgical treatment and non-surgical treatment.^[6]^ Non-surgical treatment: drug therapy is generally used, but there is no specific drug for this disease, only analgesia and anti-inflammatory treatment; Or traction, physical therapy; The treatment course is long and ineffective. Surgical treatment: Some patients will adopt surgical treatment, but the operation is risky and needs to be performed several times.^[7]^ Most patients can relieve or relieve clinical symptoms in the short term, but the recurrence rate is high, the long-term effect is poor, and there are some adverse reactions. According to the classification of disease differentiation in Traditional Chinese medicine, primary sciatica belongs to the category of “bi disease” and “lumbago and leg pain” in Chinese medicine. As early as in Lingshu • Zhou Bi, sciatica was recorded: “Cold and damp air can be separated between inside and outside flesh”, and the pain “follows the pulse above and below, and cannot be left or right”. The academic circles describe the etiology and pathogenesis of this disease as wind-cold and dampness evil, invading the human body by deficiency, causing obstruction of qi and blood, blocking of channels and collaterals or falling down by trauma, causing qi and blood disorder, and causing diseases due to obstruction of channels and collaterals.^[8]^ The main site of the disease is the part of the foot yangming meridian and the foot sun meridian. Therefore, the traditional Chinese medicine treatment of primary sciatica is generally the method of warming the meridian to dissipate cold, dispel wind and dehumidify, and activate blood circulation.

### 1.2 Description of the intervention

As an important part of Chinese medicine,^[9,10]^ acupuncture and moxibustion is simple, effective, light and convenient, which can quickly relieve the pain of patients.^[10]^ It covers acupuncture, moxibustion, cupping, blood pricking and other methods. Warm acupuncture,^[11]^ refers to the moxa roll or moxa cone pointing to the heat of combustion.^[12]^ As the needle body is introduced into the body inside, primary sciatica patients mostly are empty outside is not solid, again by pathogens such as cold and dampness, warm acupuncture moxibustion and acupuncture method, as well as to reinfor ceinternal centering, cold WenTong meridian, dehumidification, eliminating stasis blood, pain fights, and so on.

Warm acupuncture therapy has a significant effect on primary sciatica, which is easy to use, safe and reliable, and has no toxic side effects. The purpose of this review is to summarize clinical studies on the treatment of Primary sciatica with warm acupuncture, and the results of this review will be reliable in the presence of clinical research evidence. This article reviews the effects of warm acupuncture on primary sciatica and does not discuss other effective treatments.

### 1.3 The reason to perform this overview

The purpose of this study was to systematically review the existing clinical literature to evaluate the efficacy and safety of warm acupuncture therapy in the treatment of primary sciatica.^[13]^

## 2 Method

### 2.1 Registration

The protocol has been registered on the International Platform of Registered Systematic Review and Meta-analysis Protocols (INPLASY) (registration number, INPLASY2020120109; https://inplasy.com/?s=2020120109) basing on the Preferred Reporting Items for Systematic Reviews and Meta-Analyses Protocols (PRISMA-P) statement guidelines.^[14]^

### 2.2 Inclusion criteria for study selection

#### 2.2.1 Types of studies

Only RCTs will be included; quasi-RCTs and randomized cross-over studies will be excluded. Blinding will not be considered because of the characteristics of Warm acupuncture therapy (Table 1.).

**Table 1:** Search Strategy (PubMed).

Order	Strategy
#1	Search “ Primary sciatica” [Mesh] Sort by: Publication Date
#2	Search ((Primary sciatica [Title/Abstract]) OR ischiatitis [Title/Abstract]) Sort by: Publication Date
#3	#1 OR #2
#4	Search (((((((randomized controlled trial [Publication Type]) OR controlled clinical trial [Publication Type]) OR randomized [Title/Abstract]) OR drug therapy [MeSH Subheading]) OR placebo [Title/Abstract]) OR randomly [Title/Abstract]) OR trial [Title/Abstract]) OR groups [Title/Abstract] Sort by: Publication Date
#5	Search (humans [MeSH Terms]) NOT animals [MeSH Terms] Sort by: Publication Date
#6	#4 AND #5
#7	Search “Warm acupuncture” [Mesh] Sort by: Publication Date
#8	Search (((Warm acupuncture [Title/Abstract]) OR needle warming moxibustion [Title/Abstract]) OR fire needling [Title/Abstract]) Sort by: Publication Date
#9	#7 OR #8
#10	#3 AND #6 AND #9

#### 2.2.2 Participants

Patients with primary sciatica will be included, including those diagnosed as synonyms for primary sciatica, such as sciatic neuritis, primary spinal stenosis, and pain caused by other superficial inflammatory lesions. There are no restrictions on sex, age, severity or duration of symptoms. Patients with secondary sciatica, such as acute infection, cauda equina syndrome, lumbar disc herniation, and those without sciatica would be excluded.

#### 2.2.3 Interventions

- The therapeutic intervention applied in the experimental group is warm acupuncture. Studies using warm acupuncture in experimental group will be included regardless of the treatment length and frequency. The controlled group can be blank control, placebo or drug therapy.
- Warm acupuncture vs other drug treatment will be included.
- Warm acupuncture vs Simple acupuncture therapy.
- Warm acupuncture vs Simple moxibustion therapy.
- Warm acupuncture vs Electric acupuncture therapy.

#### 2.2.4 Primary Outcomes

- Pain intensity. Any validated measurement scales will be included (e.g., numeric rating scale, short-form McGill Pain Questionnaire (SF-MPQ)).
- Global assessment (the proportion of patients improved or cured). Secondary outcomes
- Quality of life, for example, using the Medical Outcomes Study 36-item Short Form health survey (SF-36).
- Patient satisfaction.
- Adverse effects.

### 2.3 Search strategy

#### 2.3.1 We will search the following databases

1. The Wan-Fang Database (the inception to 2020.12);
2. MEDLINE (the inception to 2020.12);
3. EMBASE (the inception to 2020.12);
4. The Cochrane Central Register of Controlled Trials (CENTRAL; the inception to 2020.12);
5. Chinese Biomedical Literature Database (CBM; the inception to 2020.12);
6. Chinese Medical Current Content (CMCC; the inception to 2020.12);
7. China National Knowledge Infrastructure (CNKI; the inception to 2020.12).

**Figure 1:**
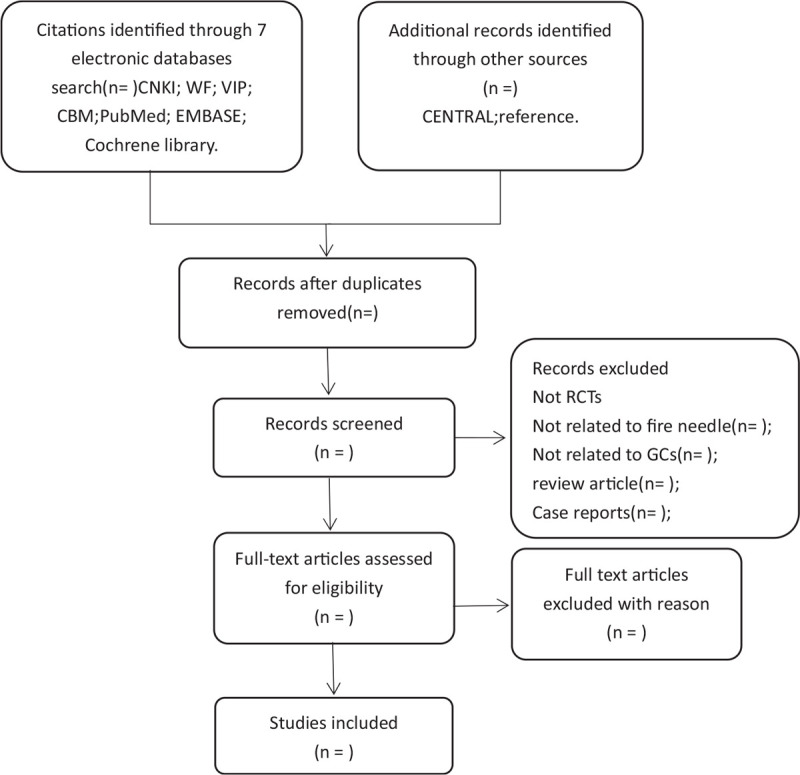
Search strategy for PubMed.

This review will use the following search terms: primary sciatica, sciatic neuritis, warm acupuncture, acupuncture, moxibustion. This study will use this strategy to search all the above databases. There is no language or type of publication restrictions. MEDLINE's search strategy can be found in supplementary online appendix 1.2.4.2. Search for other resources. Systematic review or Meta-analysis of electronic retrieval RCT. In addition, relevant medical journals and journals will be screened to identify literature that is not included in the electronic database. PubMed's initial search strategy is shown in Figure 1.

### 2.4 Selection of studies

We will select a randomized controlled trial comparing the efficacy and safety of warm acupuncture in the treatment of primary sciatica. Items belonging to one of the following items will not be included (shown in Table 1.):

1. Repeat.
2. Participants do not meet the diagnostic criteria for primary sciatica or are unknown.
3. There were no randomized controlled trials.
4. In these studies, participants did not receive a combination of fire needle therapy and traditional therapy as the main intervention,
5. This intervention includes any other traditional Chinese medicine therapy,
6. The data required is incomplete.

Eligible studies will be evaluated by the 2 authors.

### 2.5 Data extraction and management

#### 2.5.1 Assessment of risk of bias in included studies

The risk of bias assessment will be based on the Cochrane Collaboration Risk of Bias Tool.26^[15]^ Two authors (FC and CG) will independently evaluate methodological quality using the following 7 domains: random sequence generation, allocation concealment, blinding of participants and personnel, blinding of outcome assessment, incomplete outcome data, selective reporting and other bias. Other sources of bias may be caused by the different types of needles used, the duration of sciatica, the length of therapy and the age of patients. Taking these domains into account, each trial will be categorized into low risk, high risk and unclear risk. Any disagreements will be discussed and resolved by a third author (XWH).

#### 2.5.2 Measures of treatment effect

For continuous variables, we will use mean difference to evaluate the extracted data. For dichotomous variables, rate ratio (RR) will be applied to analyze. The confidence intervals (CIs) for both continuous and dichotomous variables will be set to 95%.

#### 2.5.3 Dealing with missing data

The listed corresponding author will be contacted to try and obtain any missing information from their trial. If it is impossible to obtain the data, the study will be excluded from the data synthesis.

#### 2.5.4 Assessment of heterogeneity

The following subgroup analyses will be conducted to assess the heterogeneity of the studies:

- Comparison between warm acupuncture and moxibustion therapy and simple acupuncture therapy.
- Comparison of warm acupuncture therapy and simple moxibustion therapy.

#### 2.5.5 Assessment of reporting bias

A funnel plot will be used to assess the reporting biases if 10 or more trials are included in a meta-analysis.

#### 2.5.6 Data synthesis and subgroup analysis

If meta-analysis can be conducted, RevMan V.5.3.4 software will be used to combine the RR with 95% CIs for dichotomous outcomes and the weighted mean difference or standardized mean difference with 95% CIs for continuous data. If the result of the test for heterogeneity results in *P* > .1, the fixed-effect model will be used to combine the data; if *P* < .1, the random-effect model will be used. If the data will not be suitable for combining quantitatively, in this condition, a systematic narrative synthesis will be provided with the information that presented in the text to summarise and explain the characteristics and findings of the included studies.

#### 2.5.7 Sensitivity analysis

If heterogeneity is high, sensitivity analysis will be performed according to study type, sample size, and method quality.

#### 2.5.8 Grading the quality of evidence

We will evaluate the quality of evidence and rate it into 4 levels: high, moderate, low, or very low in accordance with the Recommendations Assessment, Development and Evaluation (grade) guide lines.^[15]^

#### 2.5.9 Ethics and dissemination

Ethical recognition will not be applied because the data we extract does not involve any personal privacy. We will publish this study in a peer-reviewed journal or conference to evaluate the efficacy and safety of warm acupuncture therapy for primary sciatica.

## 3 Discussion

Primary sciatica is still a difficult disease to treat,^[16]^ although there are many treatment methods, but each has its advantages and disadvantages. In general, conservative treatment is still the main treatment method for primary sciatica.^[16,17]^ Some patients receive surgical treatment with obvious short-term efficacy, but the operation may damage the stability of the spine.^[17]^ Warm acupuncture therapy, based on the improvement of traditional acupuncture therapy, is a new way to treat diseases.^[18]^ Warm acupuncture therapy refers to the stimulation of deep acupuncture points and tissues through the conduction of warm and thermal effect of needles that have penetrated into the human body to obtain qi. The meridian acupoints of the human body are a complex system,^[19]^ which contains a wealth of nerve receptors, collagen fibers, whose main purpose is to transmit information and energy, and mast cells and other substances used to regulate the body's immunity.

Combination of moxibustion and acupuncture treatment is called - warm acupuncture; It has the functions of warming up and dispelling cold, eliminating phlegm and blood stasis, relieving swelling and pain, dredgying meridians and collaterals.^[20]^ The function improves the blood circulation, promotes the local tissue nutrition obstacle, the treatment effect is obvious, the acupuncture and moxibustion combination therapy, has the very great value, the application treatment in this disease, has the broad research prospect.

Warm acupuncture and moxibustion is a treatment method combining acupuncture and moxibustion,^[21]^ which makes use of the warm effect of acupuncture and moxibustion and the heat conduction effect of the needle body. After the acupuncture treatment of qi of the shank or alignment has been burning moxa cone into the needle, and through the infrared thermal radiation stimulate the core of moxa cone, passed by needles, permeate the body acupoints of acupoints temperature sensors and more sleep feeling appliance correlation, by 1 or 2 of reaction, achieve WenTong meridian, eliminating stasis fights, regulation the zang-fu function.^[22]^ Accelerate blood circulation and metabolism, promote immune system stress response.^[22,23]^ To our knowledge, this will be the first systematic review and meta-analysis of the efficacy and safety of warm acupuncture in the treatment of primary sciatica, it is hoped that this review will provide rigorous and objective evidence on the efficacy and safety of warm acupuncture in the treatment of Primary sciatica. However, there are limitations to this systematic review that may affect the conclusions drawn. First, the included trials were only published in English or Chinese, which may have limited the search for potential studies. Second, there may be a risk of heterogeneity among participants of different ages and genders.

In future clinical studies in this field, the criteria for diagnosis, inclusion and exclusion should be unified, and internationally recognized indicators should be selected. In the selection of sample size, we should expand the selection of sample size, and in the random allocation, we must conceal the grouping, so as to reduce the error caused by subjective consciousness in the test results. It is expected that there will be more randomized controlled trials with rigorous methodology, professional evaluation indexes and long-term follow-up in the future, so as to provide high-quality research evidence for warm acupuncture treatment of primary sciatica.

## 4 Strengths and limitations of this study

- While the curative effect of conservative treatment for primary sciatica is not very impressive, warm acupuncture may be an effective alternative therapy. As far as literature review is concerned, there is no systematic review on the treatment of sciatica by warm acupuncture published in English at present. The results of this systematic review will help clinicians make decisions regarding primary sciatica and patients seek further treatment options.
- One limitation of this systematic review is that, because of the language barrier, it can only include trials in 2 languages. As a result, relevant studies published in other languages may not be available.
- Another limitation is that the different acupuncture methods included in the study can lead to significant differences.

## Author contributions

All authors have read and approved the publication of the protocol.

**Conceptualization:** FanLei Meng.

**Data curation:** Fang Cao.

**Formal analysis:** Fang Cao, Cui Guo.

**Investigation:** Cui Guo, JingWei Wang, RuiLin Zeng, LunBin Lu, FanLei Meng, Fen Zhao.

**Methodology:** Xing-Wei He, LunBin Lu.

**Resources:** JingWei Wang.

**Software:** ChangKang Wang.

**Supervision:** Xing-Wei He.

**Validation:** RuiLin Zeng.

**Writing – original draft:** Fang Cao, Cui Guo.

**Writing – review & editing:** Xing-Wei He.

## Corrections

Fenfen Zhao's name appeared incorrectly as Fen Zhao and has since been corrected.

The funding name of The National Natural Science Foundation of China appeared incorrectly as The National Natural Science Foundation of Jiangsu Province and has since been corrected.
